# PHYSICAL ACTIVITY, BALANCE, AND BICYCLING IN OLDER ADULTS

**DOI:** 10.1093/geroni/igac059.2883

**Published:** 2022-12-20

**Authors:** Maya Baughn, Victor Arellano, Brieanna Hawthorne-Crosby, Amanda Grimes, Joey Lightner, Gregory King

**Affiliations:** University of Missouri-Kansas City, Kansas City, Missouri, United States; University of Missouri-Kansas City, Kansas City, Missouri, United States; University of Missouri-Kansas City, Kansas City, Missouri, United States; University of Missouri-Kansas City, Kansas City, Missouri, United States; University of Missouri-Kansas City, Kansas City, Missouri, United States; University of Missouri-Kansas City, Kansas City, Missouri, United States

## Abstract

Falls are a critical public health issue among older adults. One notable factor contributing to falls in older adults is a deterioration of the structures supporting balance and overall balance control. Preliminary evidence suggests older adults who ride a bicycle have better balance than those who do not. Cycling may be an effective intervention to prevent falls among older adults. This study aims to objectively measure the relationship between bicycling, physical activity, and balance for older adults. Older adult cyclists (n=19) and non-cyclists (n=27) were recruited to (1) complete a survey that assessed demographics; (2) wear an accelerometer for 3 weeks to objectively assess physical activity; and (3) complete balance-related tasks on force platforms. Mann-Whitney U-tests were performed to understand differences in balance and physical activity between cyclists and non-cyclists. Cyclists are significantly more physically than non-cyclists (U = 102.00, p = .015; U = 81.00, p=.002). Cyclists had less sway velocity in eyes open conditions than non-cyclists (U = 360.00, p=.009), indicating a more tightly regulated postural control strategy that may relate to higher stability in cyclists. Task duration among cyclists in single-leg stand was longer than non-cyclists (left leg: p < .001 and right leg: p < .005) indicating increased postural stability. This study demonstrates the possible implications for cycling on balance and reducing fall risk.

